# Supplementary material for: Deduction of signaling mechanisms from cellular responses to multiple cues
Source: NPJ Syst Biol Appl. 2022 Nov 30;8:48. doi: 10.1038/s41540-022-00262-5 (PMC9712676; doi:10.1038/s41540-022-00262-5)
Supplement: Supplementary file 1 — Supplementary Information [file 41540_2022_262_MOESM1_ESM.pdf]

Supplementary Information for "Deduction of signaling mechanisms from cellular responses to multiple cues"

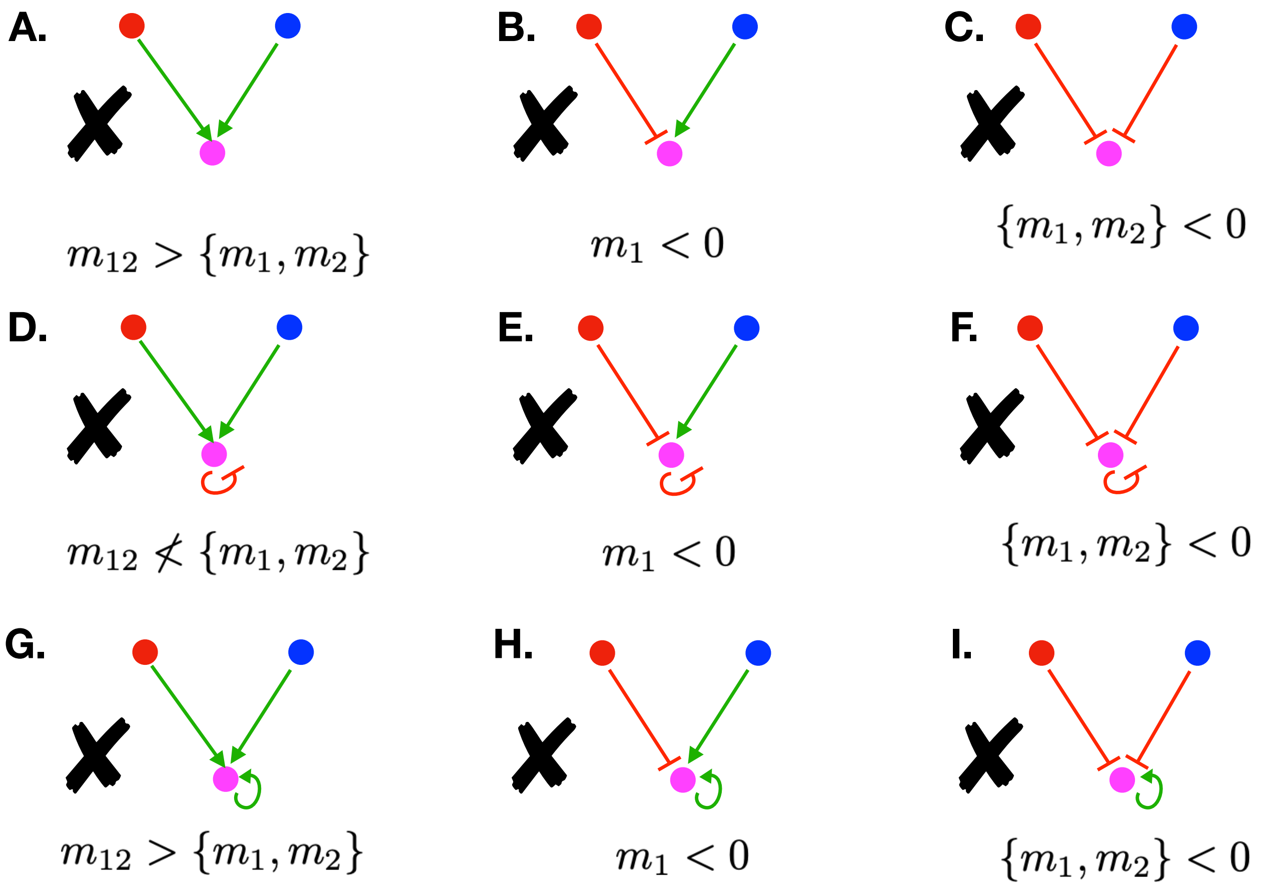

Supplementary Figure 1: **Slope antagonism with three-node regulatory networks.** Accounting for symmetry there are nine three-node regulatory networks. No three node network can satisfy the slope antagonism condition. The reasons for not satisfying the slope antagonism condition are given under each network. Here, $m_{12} = \partial m(s_1, s_2)/\partial s_1 + \partial m(s_1, s_2)/\partial s_2, m_1 = \partial m(s_1, 0)/\partial s_1, m_2 = \partial m(0, s_2)/\partial s_2$. Calculations are performed in Mathematica (see Code Availability in the main text).

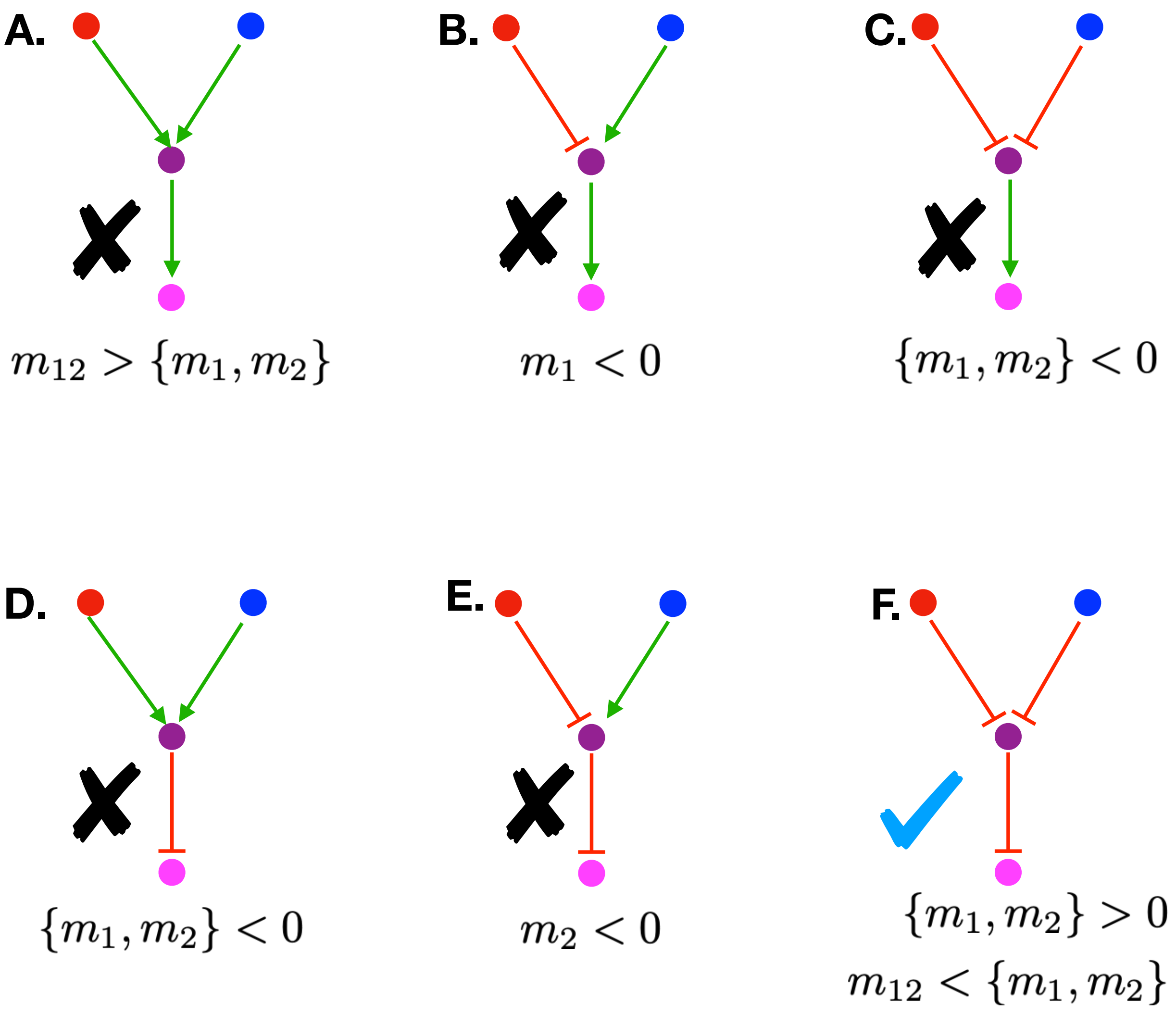

Supplementary Figure 2: **Slope antagonism with four-node regulatory networks.** Accounting for symmetry there are six four-node regulatory networks with the minimum of three edges. Only one, F, can satisfy the slope antagonism condition. The reasons for satisfying or not satisfying the slope antagonism condition are given under each network. Here, $m_{12} = \partial m(s_1, s_2)/\partial s_1 + \partial m(s_1, s_2)/\partial s_2$, $m_1 = \partial m(s_1, 0)/\partial s_1$, $m_2 = \partial m(0, s_2)/\partial s_2$. Calculations are performed in Mathematica (see Code Availability in the main text).

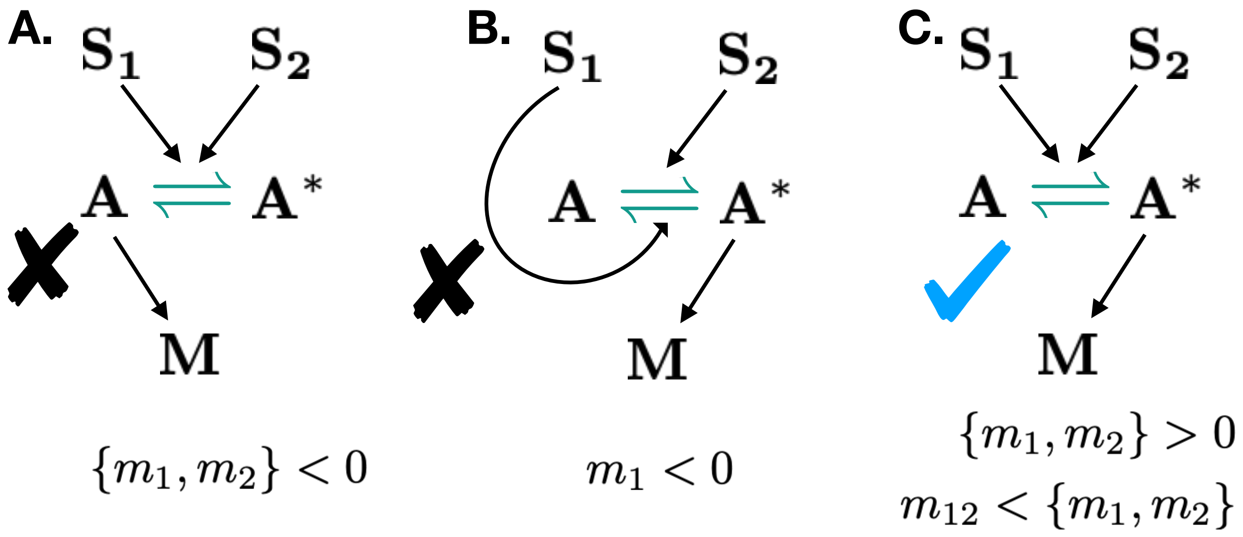

Supplementary Figure 3: **Slope antagonism with conversion networks.** Accounting for symmetry there are three conversion networks with the minimum of five nodes and three edges. Only one, C, can satisfy the slope antagonism condition. The reasons for satisfying or not satisfying the slope antagonism condition are given under each network. Here, $m_{12} = \partial m(s_1, s_2)/\partial s_1 + \partial m(s_1, s_2)/\partial s_2, m_1 = \partial m(s_1, 0)/\partial s_1, m_2 = \partial m(0, s_2)/\partial s_2$. Calculations are performed in Mathematica (see Code Availability in the main text).

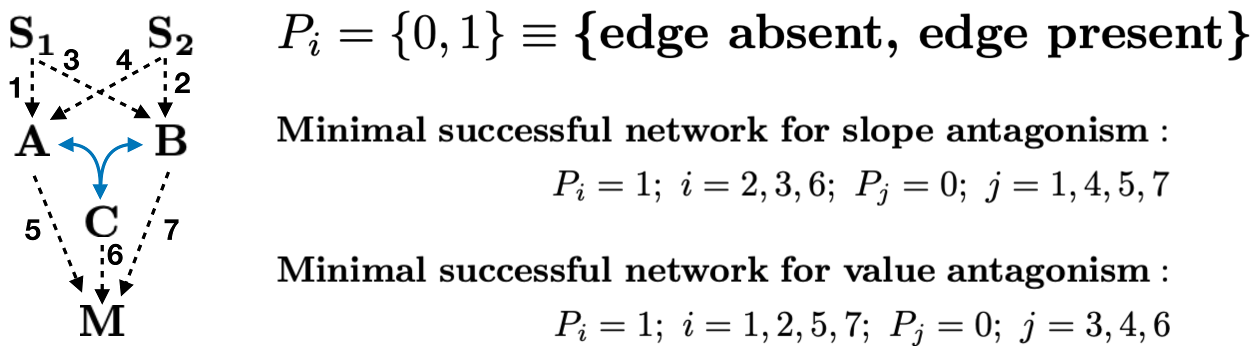

Supplementary Figure 4: **Six-node binding networks.** There are $2^7 = 128$ six-node binding networks, depending on the presence or absence of the seven edges. The minimal network that can satisfy the slope antagonism condition has three edges (2, 3, and 6) and is shown in Fig. 4F in the main text. The minimal network that can satisfy the value antagonism condition has four edges (1, 2, 5, and 7) and is shown in Fig. 5F in the main text. Calculations are performed in Mathematica (see Code Availability in the main text).

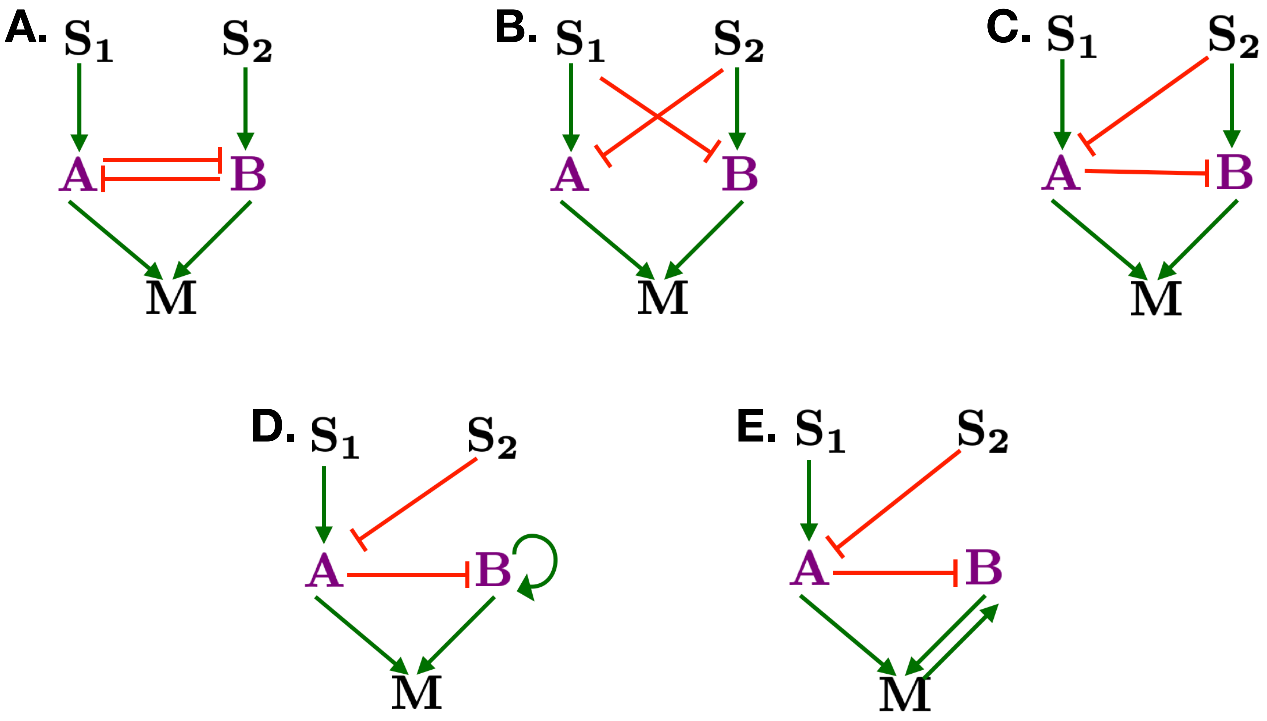

Supplementary Figure 5: **Value antagonism with regulation networks.** Five unique five-node, six-edge regulation networks are found using the our mathematical framework to be capable of showing value antagonism. The top three exhibit mutual inhibition. Calculations are performed in Mathematica (see Code Availability in the main text).

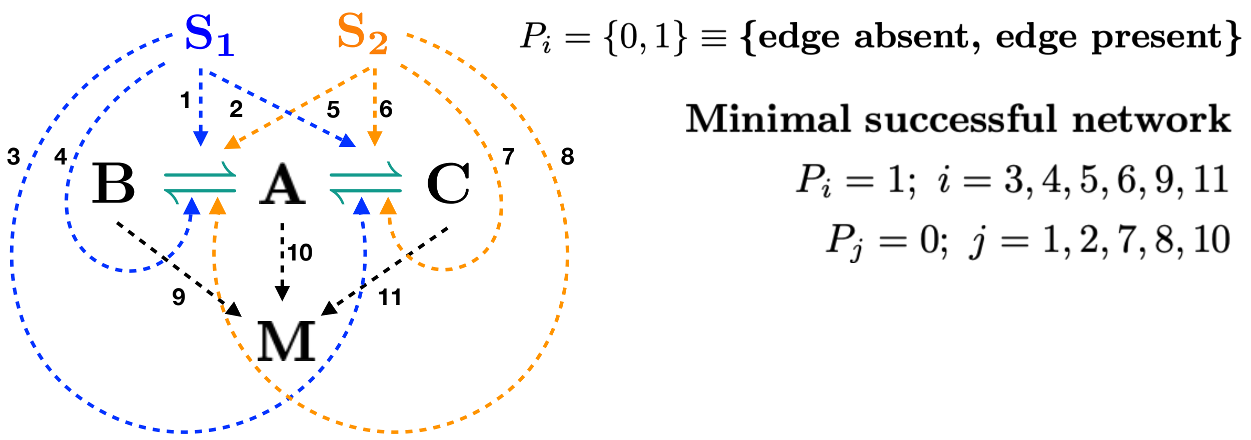

Supplementary Figure 6: **Value antagonism conversion networks.** There are $2^{11} = 2{,}048$ six-node conversion networks, depending on the presence or absence of the eleven edges. The minimal network that can satisfy the value antagonism condition has six edges (3, 4, 5, 6, 9, and 11) and is shown in Fig. 5D in the main text. Calculations are performed in Mathematica (see Code Availability in the main text).
